# *N*-Acetylcysteine and Ceftriaxone as Preconditioning Strategies in Focal Brain Ischemia: Influence on Glutamate Transporters Expression

**DOI:** 10.1007/s12640-016-9602-z

**Published:** 2016-02-09

**Authors:** Weronika Krzyzanowska, Bartosz Pomierny, Boguslawa Budziszewska, Malgorzata Filip, Joanna Pera

**Affiliations:** Department of Biochemical Toxicology, Faculty of Pharmacy, Jagiellonian University, Medical College, Medyczna 9, 30-688 Krakow, Poland; Department of Toxicology, Faculty of Pharmacy, Jagiellonian University, Medical College, Medyczna 9, 30-688 Krakow, Poland; Department of Neurology, Jagiellonian University, Medical College, Botaniczna 3, 31-503 Krakow, Poland

**Keywords:** Brain ischemia, Brain preconditioning, *N*-acetylcysteine, Ceftriaxone, Neuroprotection

## Abstract

Glutamate (Glu) plays a key role in excitotoxicity-related injury in cerebral ischemia. In the brain, Glu homeostasis depends on Glu transporters, including the excitatory amino acid transporters and the cysteine/Glu antiporter (xc-). We hypothesized that drugs acting on Glu transporters, such as ceftriaxone (CEF, 200 mg/kg, *i.p.*) and N-acetylcysteine (NAC, 150 mg/kg, *i.p.*), administered repeatedly for 5 days before focal cerebral ischemia in rats and induced by a 90-min middle cerebral artery occlusion (MCAO), may induce brain tolerance to ischemia. We compared the effects of these drugs on brain infarct volume, neurological deficits and the mRNA and protein expression of the Glu transporter-1 (GLT-1) and xc- with the effects of ischemic preconditioning and chemical preconditioning using 3-nitropropionic acid. Administration of CEF and NAC significantly reduced infarct size and neurological deficits caused by a 90-min MCAO. These beneficial effects were accompanied by changes in GLT-1 expression caused by a 90-min MCAO at both the mRNA and protein levels in the frontal cortex, hippocampus, and dorsal striatum. Thus, the results of this study suggest that the regulation of GLT-1 and xc- plays a role in the development of cerebral tolerance to ischemia and that this regulation may be a novel approach in the therapy of brain ischemia.

## Introduction

Excitotoxicity related to excessive glutamate (Glu) release plays an important role in the pathophysiology of brain ischemia (Globus et al. 1991). The glial high-affinity glutamate transporter 1 (GLT-1) and the cysteine/Glu antiporter xc- cooperate to actively regulate the concentration of extracellular Glu (Danbolt 2001; Sato et al. 1999). Brain ischemia is accompanied by a massive release of Glu; thus, the role of both GLT-1 and xc- in this phenomenon is important. Moreover, modulation of the glutaminergic system is also associated with ischemic preconditioning (IP). In this process, the brain develops a tolerance to severe, damaging ischemia by previous exposure to low-intensity and potentially damaging agents such as short-term ischemia. Transient, but deleterious, global brain ischemia decreased GLT-1 protein expression (Raghavendra Rao et al. 2000), whereas IP upregulated GLT-1 expression (Liu et al. 2011) and reduced an ischemia-induced Glu release (Romera et al. 2007). Xc- has been implicated in the release of Glu in exchange with cystine from astrocytes and microglia and thereby in the induction of neuronal cell death by excitotoxicity (Piani and Fontana 1994).

These findings suggest that the pharmacological modulation of GLT-1 and xc- could have important therapeutic implications. β-lactam antibiotics, including ceftriaxone (CEF), upregulate GLT-1 expression and present neuroprotective effects (Rothstein et al. 2005). Intraperitoneal (*i.p.*) administration of CEF for 5 consecutive days prior to 90 min of a middle cerebral artery occlusion (MCAO) reduced infarct volume and neurological deficits in rats (Chu et al. 2007). Substances that affect xc- can also attenuate the Glu excitotoxicity. For example, *N*-acetylcysteine (NAC) is known to modulate the neuroprotective effects of xc- (Lewerenz et al. 2013) in the brain ischemia model (Sekhon et al. 2003).

We sought to investigate the neuroprotective effects of CEF and NAC in the MCAO brain ischemia model and their influence on the expression of GLT-1 and xc-. This is the first report that reveals the influence of NAC and 3-nitropropionic acid (3NP) on these Glu transporters in the 90 min MCAO model. In addition, the comparison of pharmacological interventions with the reference preconditioning strategies (IP or 3NP) described in this study is also an innovative approach.

## Materials and Methods

### Animals

All experiments were performed on male *Wistar* rats (280–320 g; Charles River, USA). The animals were kept on a normal day–night cycle at 22 ± 2 °C with free access to food and water. The study was carried out in accordance with the Guide for the Care and Use of Laboratory Animals published by the National Institutes of Health and was approved by the First Local Ethical Committee on Animal Testing at the Jagiellonian University in Krakow (permit: 78/2011). All studies involving animals are reported according to the ARRIVE (Animal Research: Reporting of In Vivo Experiments) guidelines, including the blinding procedure of animal identities on each level of experiments.

### Drugs and Experimental Design

CEF (Biotrakson, Polpharma, Poland), NAC (Sigma Aldrich, USA), and 3NP (Sigma Aldrich, USA) were dissolved in saline (the NAC and 3NP solutions were neutralized with 10 % NaOH solution). Rats either received CEF (200 mg/kg), NAC (150 mg/kg), or saline *i.p.* for 5 consecutive days. 3NP was administered in a single dose of 20 mg/kg *i.p.* The injection volume was 1 µL/g of body weight.

Three days after the last injection (saline, CEF, NAC, or 3NP), 90-min MCAO or sham surgery was performed. The animals were randomly assigned to the following groups: sham surgery (SHAM), 90-min MCAO (ISCH), 20-min MCAO 3 days prior to sham operation (IP), 20-min MCAO 3 days prior to 90-min of occlusion (IP + ISCH), NAC administration 3 days prior to sham operation (NAC), NAC administration prior to 90-min MCAO (NAC + ISCH), CEF administration 3 days prior to sham operation (CEF), CEF administration 3 days prior to 90-min MCAO (CEF + ISCH), 3NP administration 3 days prior to sham operation (3NP) and 3NP administration 3 days prior to 90-min MCAO (3NP + ISCH). Eight animals from each group were dedicated to the real-time PCR analysis, eight rats for the ELISA assay and eight for the evaluation of the brain ischemia infarct volume by TTC staining. All animals underwent a neurological assessment.

### Focal Cerebral Ischemia Model

Transient focal cerebral ischemia was induced in anesthetized rats by an intraluminal filament occlusion of the left middle cerebral artery (MCA) according to the Koizumi method using a silicone-coated filament (Doccol Corp., USA) (Koizumi et al. 1986). The identity of the animal was blinded to the operator. All surgical procedures were conducted under a stereoscopic surgical microscope (PZO Warszawa, Poland), and the physiological body temperature was maintained with a heating blanket (Harvard Apparatus, UK). The effectiveness of the occlusion was determined using a blood flowmeter (Perimed, Sweden). Anesthesia was induced by a ketamine/xylazine (Ketamina, Sedazin, Biowet-Puławy, Poland) mixture (3/1, v/v, respectively) administered *i.p.* Anesthetics were given in 300 µL injections, containing 75 mg/kg of ketamine and 5 mg/kg of xylazine by body weight.

The occlusion times were 20 and 90 min for IP and ISCH, respectively. After the occlusion, the filament was withdrawn to restore blood flow. Sham operations were carried out as a MCAO except for the insertion of the filament.

### Neurological Evaluation

Functional assessments of animals were conducted with the identities blinded to the observer and performed 24 h after surgery using the Philips grading system (Phillips et al. 2000). Neurobehavioral findings were scored on a 10 point scale where 0 indicated no neurological deficit and 10 points indicated a maximal neurological deficit.

### Evaluation of the Infarct Volume by TTC Staining

Rats were killed 24 h after reperfusion by decapitation. Brains were immediately removed and sliced using a brain matrix (Harvard Apparatus, USA). Coronal sections (2 mm thick) were stained with a 1 % solution of 2,3,5-triphenyltetrazolium chloride (TTC) dye (Sigma Aldrich, USA) in 0.01 M phosphate-buffered saline (Sigma Aldrich, USA) at 37 °C for 10 min in the dark. Sections were fixed in 10 % phosphate-buffered formalin (Sigma Aldrich, USA) overnight at 4 °C. Brain slices were photographed under the surgical microscope (PZO Warszawa, Poland) equipped with a digital camera (Optica, Italy) by an investigator blinded to subject identity. The infarct area was determined by NIH ImageJ software (National Institutes of Health, version 8.0) by the same investigator. The infarct volume was calculated as a sum of each outlined white area multiplied by the thickness of a brain section and expressed in mm^3^.

### Real-Time PCR

Rats were decapitated 24 h after reperfusion; the brains were immediately removed and selected brain structures, the frontal cortex, hippocampus, and dorsal striatum, were isolated and immersed in *RNAlater* stabilization solution (Ambion, USA) for 24 h at 4 °C to preserve the RNA. The total RNA from the collected brain regions was extracted using TRI Reagent (Zymo Research, USA) and purified with the Direct-zol RNA MiniPrep Kit (Zymo Research, USA) according to the manufacturer’s protocol, including the in-column DNAse I treatment step. The isolated RNA was stored until used at −80 °C until used. Reverse transcription reactions and real-time PCR were conducted using the PrimeQ real-time PCR system (Techne, USA). The total RNA was subsequently transcribed into cDNA using the Transcriptor First Strand cDNA Synthesis Kit (Roche, USA) according to the manufacturer’s procedure. The cDNA was stored at −80 °C until used. The relative cDNA quantification of GLT-1, xCT (the light chain of xc-) and a reference gene (glyceraldehyde 3-phosphate dehydrogenase, GAPDH) was measured using the commercial TaqMan Gene Expression Assay (Applied Biosystems, USA). Real-time PCR was performed using the Fast Start Universal Probe Master (ROX) (Roche, USA) according to the manufacturer’s procedure. The expression levels of each gene were normalized to GAPDH levels; the fold change in expression was determined using the ΔΔc(t) method of relative quantification.

### Enzyme-Linked Immunosorbent Assays (ELISA)

The expression of GLT-1 and xCT proteins were determined using ELISA 96 well Assay Kits (Cloud-Clone Corp., USA). Animals were decapitated 24 h after reperfusion. Selected brain structures, the frontal cortex, hippocampus, and dorsal striatum, were isolated and immediately frozen on dry ice and stored at −80 °C. Briefly, 10 % (w/v) homogenates of brain structures were prepared in ice-cold PBS with a mechanical homogenizer (ProScientific Inc., USA) and an ultrasonic processor (Hielscher-Ultrasound, Germany). Homogenates were centrifuged at 10000×*g* for 10 min at 4 °C. The obtained supernatants were diluted 1:1 in PBS and added to 96-well plates, which were processed according to the manufacturer’s protocol. The optical density was read at a wavelength of 450 nm on a multiwell plate reader (TECAN, Switzerland).

### Statistical Analyses

All data are expressed as the mean ± SEM. The protein/mRNA expression data were analyzed using the two-way ANOVA for factors pretreatment (CEF/NAC/3NP/IP), surgery (SHAM or MCAO), and pretreatment × surgery. If statistical significance was found through analysis using ANOVAs, Tukey’s post hoc test was then conducted to test comparisons. Moreover, a priori pairwise comparisons between the ischemic and sham groups were made with Student’s *t* test. Infarction volume data were analyzed using Student’s *t* test. A Mann–Whitney test was applied for the analysis of neurological deficits. A *p* < 0.05 was considered as statistically significant.

## Results

### Neurological Deficit

**Fig. 1:**
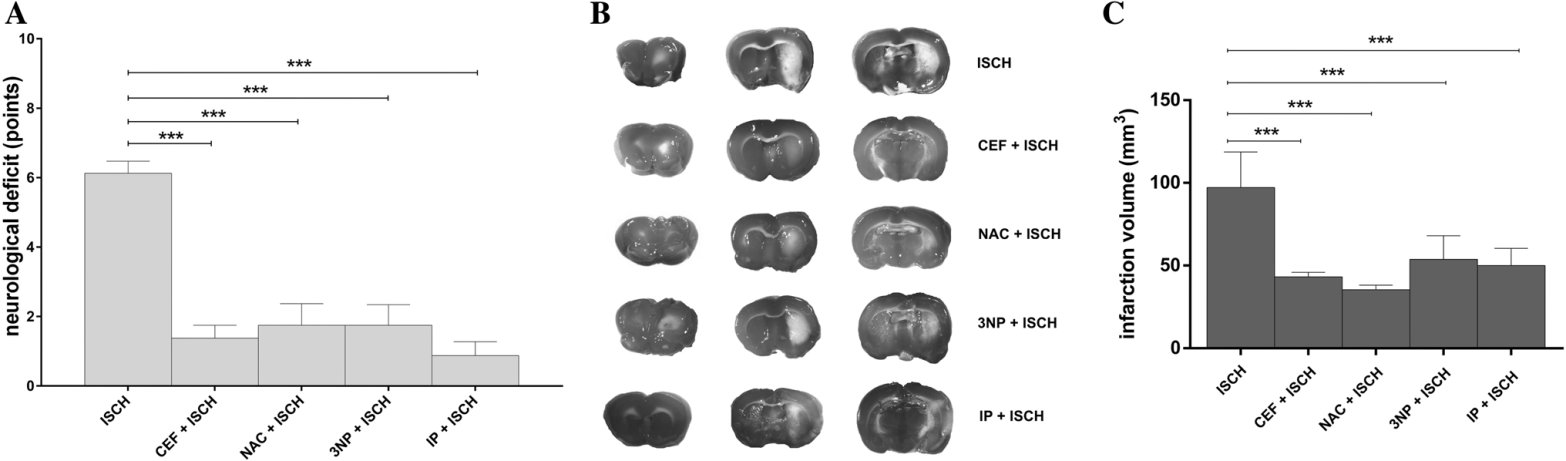
Neuroprotective effects of interventions. **a** Decreased neurological deficit 24 h after ischemia (***p < 0.001 vs. ISCH, Mann–Whitney, *n* = 24/group). **b** Representative TTC-stained brain sections and corresponding histogram **c** representing the calculated infarct volume in ISCH, CEF + ISCH, NAC + ISCH, 3NP + ISCH, and IP + ISCH groups (***p < 0.001 vs. ISCH, *t* test, *n* = 8/group) 24 h after reperfusion. *3NP* 3-nitropropionic acid, *CEF* ceftriaxone, *IP* ischemic preconditioning, *ISCH* 90 min of occlusion, *NAC* *N*-acetylcysteine, *SHAM* sham operated animals

After a 90-min MCAO, the neurological deficits were scored and received an average of 6.13 ± 0.35 points. All interventions significantly improved the neurological status. The reduction in test score when compared with a 90-min MCAO was as follows: 1.38 ± 0.38 points for CEF + ISCH (*p* < 0.001), 1.75 ± 0.62 points for NAC + ISCH (*p* < 0.001), 2.38 ± 0.96 for 3NP + ISCH (*p* < 0.001), and 0.63 ± 0.38 points for IP + ISCH (*p* < 0.001) (Fig. 1a). Animals that were only subjected to preconditioning interventions showed no neurological deficits.

### Infarct Volume

Pretreatment with CEF, NAC, 3NP, or IP prior to a 90-min MCAO significantly reduced the infarct volume by 53.97, 61.65, 43.34, and 47.01 mm^3^, respectively. The mean infarct volumes were 43.18 ± 2.78 mm^3^ for CEF + ISCH (*t*[14] = 6.67, *p* = 0.001), 35.50 ± 0.95 mm^3^ for NAC + ISCH (*t*[14 = 8.05], *p* = 0.001), 53.81 ± 5.04 mm^3^ for 3NP + ISCH (*t*[14] = 4.75, *p* = 0.001) and 50.1 ± 3.66 mm^3^ for IP + ISCH (*t*[14] = 5.58, *p* = 0.001) vs. 97.15 ± 7.60 mm^3^ for ISCH (Fig. 1b, c). Preconditioning interventions without a subsequent 90 min MCAO did not produce brain infarction.

### GLT-1 mRNA Expression

**Fig. 2:**
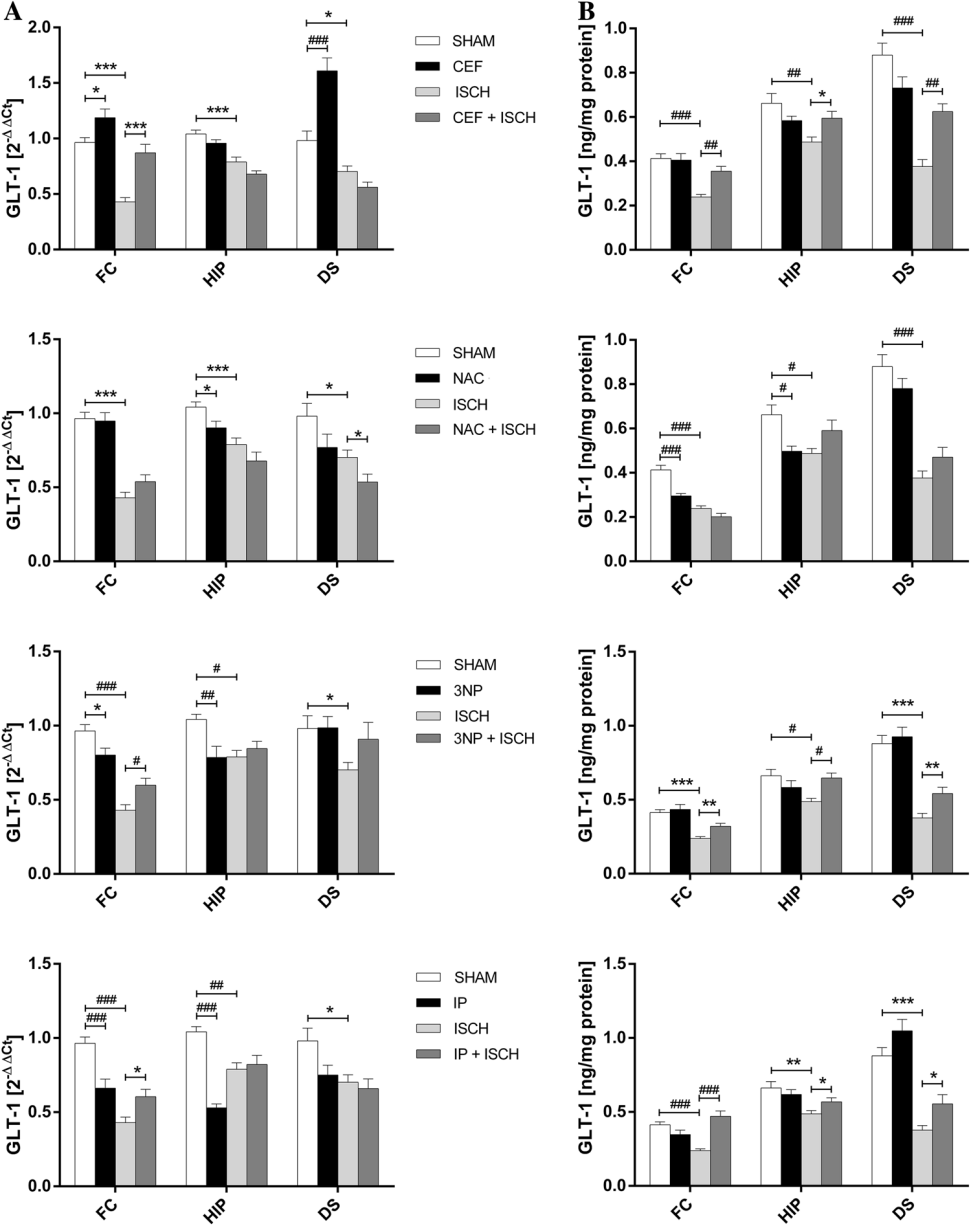
Influence of interventions on the expression of mRNA (**a**) and protein (**b**) of GLT-1 in the frontal cortex (FC), hippocampus (HIP) and dorsal striatum (DS) in the following groups: SHAM, ISCH, CEF, CEF + ISCH, NAC, NAC + ISCH, 3NP, 3NP + ISCH, IP + ISCH, and IP (*p < 0.05, **p < 0.01, ***p < 0.001 vs. ISCH or SHAM, Student’s *t* test, ^#^ *p* < 0.05, ^##^ *p* < 0.01, ^###^ *p* < 0.001 vs. ISCH or SHAM, Two-way ANOVA, Post hoc Tukey’s test, *n* = 8/group) 24 h after reperfusion. *3NP* 3-nitropropionic acid, *CEF* ceftriaxone, *IP* ischemic preconditioning, *ISCH* 90 min of occlusion, *NAC* *N*-acetylcysteine; *SHAM* sham operated animals

In the ISCH group, the GLT-1 mRNA levels were significantly decreased in the frontal cortex (*t*[14] = 10.30, *p* = 0.001), hippocampus (*t*[14] = 4.52, *p* = 0.002), and dorsal striatum (*t*[14] = 2.81, *p* = 0.014) (Fig. 2a).

A two-way ANOVA revealed that CEF alone enhanced GLT-1 expression in the dorsal striatum (*p* < 0.001). A priori comparisons with Student’s *t* test also showed a significant increase of GLT-1 mRNA in the frontal cortex (*t*[14] = 2.94, *p* = 0.026). Moreover, a priori Student’s *t* test showed that CEF pretreatment in ischemic rats significantly elevated the GLT-1 mRNA levels in the frontal cortex (*t*[14] = 5.13, *p* = 0.001) (Fig. 2a; Table 1).

**Table 1:** Table of factors interaction

2-way ANOVA factor	Transporter	Brain structure FC		HIP		DS	
		Expression level mRNA	Protein	mRNA	Protein	mRNA	Protein
CEF							
Pretreatment × surgery	GLT-1	*F*(1,28) = 3.09, *p* = 0.089	***F*** **(1,28)** = **8.36,** ***p*** = **0.007**	*F*(1,28) = 0.15, *p* = 0.702	***F*** **(1,28)** = **9.05,** ***p*** = **0.005**	***F*** **(1,28)** = **23.35,** ***p*** **<** **0.001**	***F*** **(1,28)** = **20.20,** ***p*** **<** **0.001**
Pretreatment × surgery	xc-	***F*** **(1,28)** = **5.5,** ***p*** = **0.026**	***F*** **(1,28)** = **13.95,** ***p*** **<** **0.001**	*F*(1,28) = 0.63, *p* = 0.432	*F*(1,28) = 1.61, *p* = 0.215	***F*** **(1,28)** = **33.41,** ***p*** **<** **0.001**	*F*(1,28) = 1.98, *p* = 0.171
NAC							
Pretreatment × surgery	GLT-1	*F*(1,28) = 1.82, *p* = 0.188	***F*** **(1,28)** = **7.08,** ***p*** = **0.013**	*F*(1,28) = 0.09, *p* = 0.766	***F*** **(1,28)** = **13.68,** ***p*** **<** **0.001**	*F*(1,28) = 0.09, *p* = 0.758	***F*** **(1,28)** = **4.65,** *p* = **0.04**
Pretreatment × surgery	xc-	***F*** **(1,28)** = **25.57,** ***p*** **<** **0.001**	*F*(1,28) = 3.29, *p* = 0.08	*F*(1,28) = 0.29, *p* = 0.594	*F*(1,28) = 0.005, *p* = 0.944	*F*(1,28) = 0.19, *p* = 0.66	***F*** **(1,28)** = **8.54,** ***p*** = **0.007**
3NP							
Pretreatment × surgery	GLT-1	***F*** **(1,28)** = **14.39,** ***p*** **<** **0.001**	*F*(1,28) = 2.95, *p* = 0.097	***F*** **(1,28)** = **8.89,** ***p*** = **0.006**	***F*** **(1,28)** = **10.11,** ***p*** = **0.004**	*F*(1,28) = 1.41, *p* = 0.245	*F*(1,28) = 2.47, *p* = 0.127
Pretreatment × surgery	xc-	***F*** **(1,28)** = **31.75,** ***p*** **<** **0.001**	*F*(1,28) = 3.03, *p* = 0.093	***F*** **(1,28)** = **24.06,** ***p*** **<** **0.001**	*F*(1,28) = 0.25, *p* = 0.62	*F*(1,28) = 2.74, *p* = 0.109	*F*(1,28) = 0.16, *p* = 0.689
IP							
Pretreatment × surgery	GLT-1	***F*** **(1,28)** = **24.52,** ***p*** **<** **0.001**	***F*** **(1,28)** = **30.76,** ***p*** **<** **0.001**	***F*** **(1,28)** = **39.49,** ***p*** **<** **0.001**	*F*(1,28) = 3.63, *p* = 0.067	*F*(1,28) = 1.94, *p* = 0.174	*F*(1,28) = 0.293, *p* = 0.592
Pretreatment × surgery	xc-	***F*** **(1,28)** = **45.62,** ***p*** **<** **0.001**	*F*(1,28) = 3.71, *p* = 0.064	***F*** **(1,28)** = **70.93,** ***p*** **<** **0.001**	*F*(1,28) = 0.11, *p* = 0.741	*F*(1,28) = 0.34, *p* = 0.564	*F*(1,28) = 0.27, *p* = 0.606

Interactions between factors: pretreatments (NAC, CEF, 3NP or IP) and surgery (SHAM or ISCH)

*NAC* *N*-acetylcysteine, *CEF* ceftriaxone, *3NP* 3-nitropropionic acid, *IP* ischemic preconditioning, *GLT-1* Glu transporter-1, *xc-* Glu/cystine antiporter

*F* values, which reached statistical significance (shown in bold) indicate groups classified for further Post hoc analysis (Tukey’s test)

A priori Student’s *t* test showed that NAC alone reduced GLT-1 mRNA expression in the hippocampus (*t*[14] = 2.44, *p* = 0.029); in the ISCH animals, NAC significantly reduced GLT-1 expression in the dorsal striatum (*t*[14] = 2.27, *p* = 0.039) (Fig. 2a; Table 1).

3NP pretreatment significantly decreased GLT-1 mRNA expression as shown by ANOVA in the hippocampus of sham-operated animals (*p* = 0.009) but increased GLT-1 mRNA in the frontal cortex of the ischemic group (*p* = 0.002). A priori Student’s *t* test revealed that 3NP alone in the intact frontal cortex significantly decreased GLT-1 mRNA expression (*t*[14] = 2.58, *p* = 0.022) (Fig. 2a; Table 1).

A two-way ANOVA revealed that IP alone significantly decreased the GLT-1 mRNA levels in the frontal cortex (*p* < 0.001) and hippocampus (*p* < 0.001). Moreover, a priori Student’s *t* test showed a significant increase in GLT-1 mRNA expression in ischemic animals subjected to prior IP in the frontal cortex (*t*[14] = 2.84, *p* = 0.013) (Fig. 2a; Table 1).

### xc- mRNA Expression

**Fig. 3:**
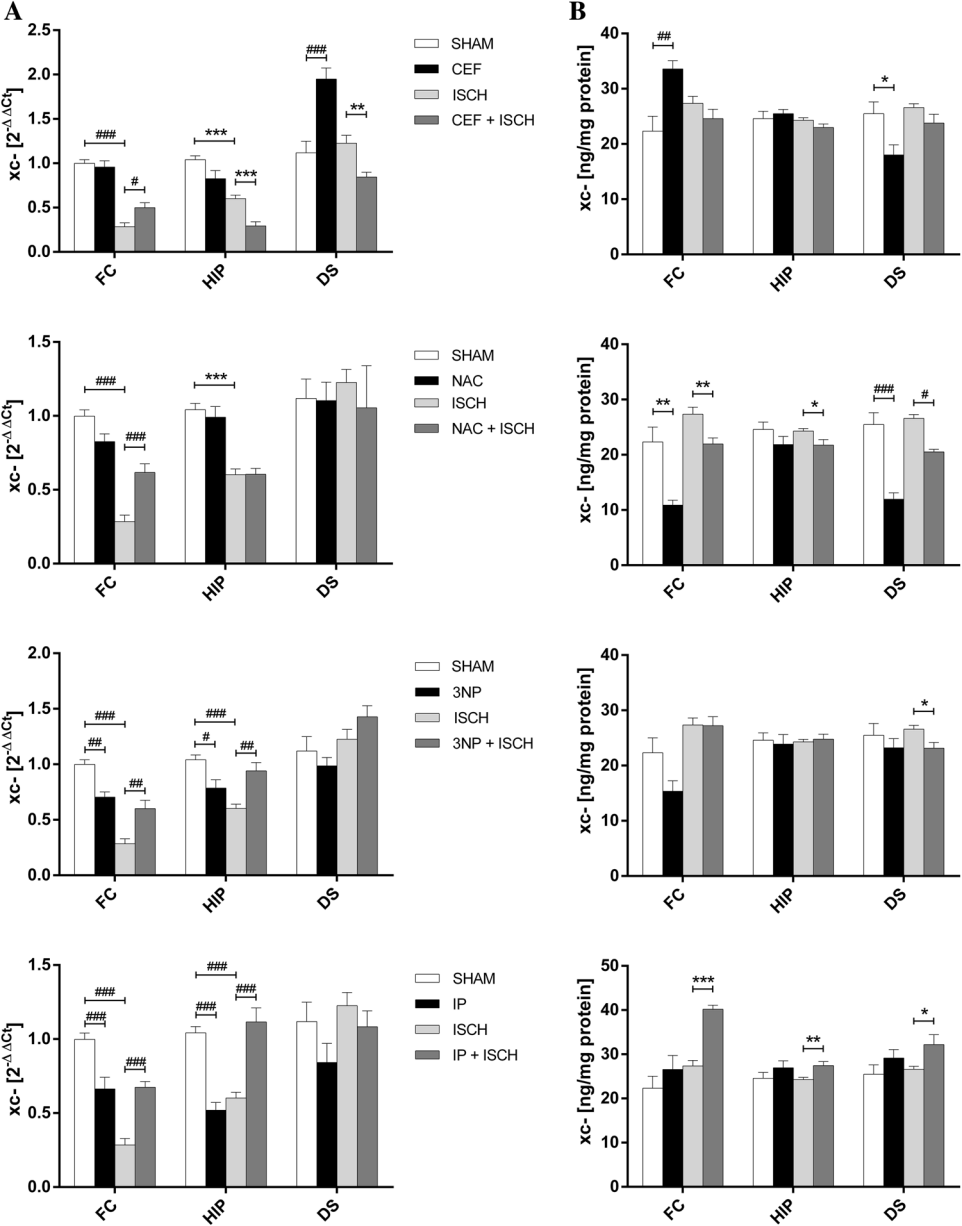
Influence of interventions on the expression of mRNA (**a**) and protein (**b**) of xc- in the frontal cortex (FC), hippocampus (HIP) and dorsal striatum (DS) in the following groups: SHAM, ISCH, CEF, CEF + ISCH, NAC, NAC + ISCH, 3NP, 3NP + ISCH, IP + ISCH, and IP (*p < 0.05, **p < 0.01, ***p < 0.001 vs. ISCH or SHAM, Student’s *t* test, ^#^ *p* < 0.05, ^##^ *p* < 0.01, ^###^ *p* < 0.001 vs. ISCH or SHAM, Two-way ANOVA, Post hoc Tukey’s test, *n* = 8/group) 24 h after reperfusion. *3NP* 3-nitropropionic acid, *CEF* ceftriaxone, *IP* ischemic preconditioning, *ISCH* 90 min of occlusion, *NAC* *N*-acetylcysteine, *SHAM* sham operated animals

In the ISCH group, xc- mRNA levels were significantly reduced in both the frontal cortex (*t*[14] = 11.62, *p* < 0.001) and hippocampus (*t*[14] = 7.59, *p* < 0.001) (Fig. 3a).

A two-way ANOVA showed that CEF alone significantly upregulated xc- mRNA expression only in the dorsal striatum (*p* < 0.001). In ischemic animals, CEF increased xc- mRNA expression in the frontal cortex (*p* = 0.044); however, a priori Student’s *t* test showed that CEF reduce xc- mRNA in the hippocampus (*t*[14] = 4.91, *p* = 0.001) and dorsal striatum (*t*[14] = 3.61, *p* = 0.003) of ischemic animals (Fig. 3a; Table 1).

Two-way ANOVA revealed that in ischemic rats, NAC administration resulted in an increase of xc- mRNA levels in the frontal cortex (*p* < 0.001) (Fig. 3a; Table 1).

The ANOVA showed that 3NP alone decreased xc- mRNA expression in the frontal cortex (*p* = 0.003) and hippocampus (*p* = 0.028). Compared with rats subjected to 90-min MCAO, 3NP administration significantly enhanced xc- mRNA expression both in the frontal cortex and in the hippocampus (*p* = 0.002 and *p* = 0.003, respectively) (Fig. 3a; Table 1).

A two-way ANOVA revealed that IP alone downregulated xc- mRNA expression in the frontal cortex and hippocampus (*p* < 0.001 and *p* < 0.001, respectively), whereas in ischemic animals, ANOVA showed that IP resulted in a significant increase of xc- mRNA in the frontal cortex and hippocampus (*p* < 0.001 and *p* < 0.001, respectively) (Fig. 3a; Table 1).

### GLT-1 Protein Expression

In the ISCH group, a significant decrease of GLT-1 protein levels was observed in the frontal cortex (*t*[14] = 7.38, *p* < 0.001), hippocampus (*t*[14] = 3.52, *p* = 0.003) and dorsal striatum (*t*[14] = 7.99, *p* < 0.001) (Fig. 2b).

In ischemic rats, a two-way ANOVA showed that CEF pretreatment upregulated GLT-1 protein expression in the frontal cortex and dorsal striatum (*p* = 0.003 and *p* = 0.002, respectively). Moreover, a priori Student’s *t* test showed an increase in GLT-1 protein expression in ischemic hippocampus (*t*[14] = 2.82, *p* = 0.014) (Fig. 2b; Table 1).

NAC alone, as revealed by ANOVA, reduced GLT-1 levels in the frontal cortex and hippocampus (*p* < 0.001 and *p* = 0.01, respectively) (Fig. 2b; Table 1).

A two-way ANOVA showed that in ischemic animals 3NP significantly raised GLT-1 protein expression in the hippocampus to SHAM levels (*p* = 0.026). Moreover, a priori Student’s *t* test indicated that in ischemic animals pretreatment with 3NP significantly increased GLT-1 expression in the frontal cortex (*t*[14] = 3.56, *p* = 0.003) and dorsal striatum (*t*[14] = 3.16, *p* = 0.007) (Fig. 2b; Table 1).

Similar results were observed in the IP groups. A two-way ANOVA revealed that in animals subjected to ischemia, IP significantly increased GLT-1 protein expression in the frontal cortex to the SHAM levels (*p* < 0.001). Moreover, a priori Student’s *t* test showed significant increases in the hippocampus (*t*[14] = 2.27, *p* = 0.04) and dorsal striatum (*t*[14] = 2.55, *p* = 0.023) (Fig. 2b; Table 1).

### xc- Protein Expression

After a 90-min MCAO, no changes in xc- protein levels were found in any of the examined structures (Fig. 3b).

As shown by two-way ANOVA, CEF alone enhanced the xc- protein expression in the frontal cortex (*p* = 0.001). Interestingly, a priori Student’s *t* test showed reduced levels of xc- protein in the dorsal striatum (*t*[14] = 2.67, *p* = 0.019) (Fig. 3b; Table 1).

For NAC only treated rats, ANOVA showed a decrease in xc- only in the dorsal striatum (*p* < 0.001). However, in the frontal cortex, as shown by Student’s *t* test, NAC administration reduced the xc- protein levels (*t*[14] = 3.99, *p* = 0.001). In ischemic rats, ANOVA showed that a prior NAC injection reduced the xc- protein expression in the dorsal striatum (*p* = 0.011). A priori Student’s *t* test revealed that NAC also reduced xc- expression in the frontal cortex (*t*[14] = 3.21, *p* = 0.006) and hippocampus (*t*[14] = 2.32, *p* = 0.036) (Fig. 3b; Table 1).

In ischemic animals, a priori Student’s *t* test showed that 3NP reduced the xc- protein expression in the dorsal striatum (*t*[14] = 2.78, *p* = 0.015) (Fig. 3b; Table 1), whereas 3NP alone did not influence protein levels in any of the structures examined.

In ISCH animals after IP, an a priori Student’s *t* test showed that higher protein levels were found in the frontal cortex (*t*[14] = 8.21, *p* < 0.001), hippocampus (*t*[14] = 3.01, *p* = 0.008) and dorsal striatum (*t*[14] = 2.37, *p* = 0.033) (Fig. 3b; Table 1). No significant changes were found after IP alone.

## Discussion

Ischemic stroke is one of the major causes of death and disability in adults. Despite significant progress in patient care, therapeutic options for stroke victims are limited, and only a small percentage of patients are treated with thrombolysis or by an endovascular approach. Thus, new therapeutic strategies are urgently needed. The induction of cerebral tolerance to ischemia is an intriguing phenomenon. It is possible to make the brain more resistant to damaging stimuli using chemical, pharmacological, or physical interventions. The molecular biology of this phenomenon is poorly understood. Glu-related excitotoxicity plays an important role in ischemic brain injury; thus, modulation of the Glu transporters is a reasonable candidate mechanism that may be involved in the induction of brain tolerance.

In the present study, we found that all four of the preconditioning strategies that were investigated, i.e., CEF, NAC, 3NP, and IP, were effective in protecting the brain against prolonged focal ischemia in a MCAO model in rats. Moreover, all of these interventions influenced the expression of the two Glu transporters that were investigated, GLT-1 and xc-.

In agreement with previous reports (Sanchez-Mendoza et al. 2010; Ketheeswaranathan et al. 2011), we observed that prolonged MCAO resulted in the decreased expression of GLT-1 at both the mRNA and protein levels in all 3 of the regions that were analyzed in the ischemic cerebral hemisphere, the dorsal striatum, the frontal cortex, and the hippocampus. The simplest mechanistic explanation for this may be that glial and neuronal cells that express GLT-1 undergo ischemia-induced cell death. Intriguingly, a 90-min MCAO caused a significant drop in xc- mRNA levels only in the frontal cortex and hippocampus. The mRNA expression in the dorsal striatum and the protein levels in all three of the investigated regions did not differ significantly when compared with non-ischemic rats. As suggested by Nurmi et al. 2004, this effect may be associated with an increase in nuclear factor-kappa B (NFκB) activity within the peri-infarct region (frontal cortex and hippocampus) followed by the suppression of the xCT gene (Nair et al. 2008). Why the mRNA and protein levels in the dorsal striatum, the region that is most severely affected by focal ischemia, did not change significantly remains unclear. This phenomenon may be related to the distribution of the cellular sources of the xc- system, which are predominantly expressed in meningeal, ependymal, and microglial cells (Massie et al. 2015). These first two types of cells were absent within the investigated striatal tissue. Activated microglia in ischemic tissue are typically present 24 h after stroke (Thiel and Heiss 2011). Thus, a lack of major cellular sources of the xc- system within the analyzed structures could result in a lack of significant changes in the transporter expression. Another potential mechanism responsible for the observed changes in xc- expression is related to the Janus protein tyrosine kinases (JAK)/signal transducers and activators (STAT) pathway. STAT3/STAT5 negatively regulates the expression of xc- (Massie et al. 2015), and the activation of STAT3 was shown in the ischemic cortex and striatum of rats subjected to transient MCAO (Justicia et al. 2000). Relatively stable protein levels in the cortex and hippocampus, despite ischemia, could result from different kinetics of mRNA and protein expression.

Not surprisingly, both pharmacological pretreatments, i.e., CEF and NAC, exerted different effects on the Glu transporters GLT-1 and xc-. Compared with untreated animals, CEF alone induced the expression of GLT-1 mRNA in the frontal cortex and striatum, but no significant changes in protein levels were found in any of the studied structures. Rothstein et al. (2005) first described the effects of CEF on GLT-1 expression; many studies have since confirmed that CEF enhances the expression of this transporter. However, depending on the experimental model, treatment paradigms, and animals used, there are several discrepancies between the published results. For instance, Thöne-Reineke et al. (2008) reported a lack of changes in GLT-1 mRNA and protein levels in the hippocampus, frontal cortex, and striatum after CEF administration, whereas Lai et al. (2011) found that in neonatal rats, CEF treatment induced GLT-1 protein levels in the cortex but not in the striatum and hippocampus. These results raise questions about the posttranscriptional regulation of GLT-1 expression in different brain regions.

In rats subjected to 90-min ischemia, a 5-day pretreatment with CEF resulted in an increased expression of GLT-1 mRNA only in the frontal cortex. In addition, the protein levels were higher in all 3 regions compared with non-preconditioned animals. These results are in agreement with findings published by Verma et al. (2010). In the frontal cortex and hippocampus, which are periinfarct areas, (both regions were largely spared in treated rats) there is an increased expression of NFκB (Schneider et al. 1999), which plays a crucial role in enhancing GLT-1 expression by CEF (Lee et al. 2008). Increased protein levels in rats pretreated with CEF and subjected to a 90-min MCAO were comparable with levels detected in the SHAM group. Since the frontal cortex and hippocampus were spared from ischemic damage, cellular loss may have been minimal or not occurring within these two regions. Whereas in the severely damaged dorsal striatum, GLT-1 upregulation in the pretreated animals may reflect an attempt to induce tolerance, but the lack of protection against ischemia, despite increased GLT-1 protein expression, could be related to the severity of ischemic damage because this area is an ischemic core region. The drop in blood flow in the dorsal striatum is the most severe of the brain regions, and this tissue dies first (Garcia et al. 1995). However, further studies to clarify the regulatory action of CEF on GLT-1 in focal cerebral ischemia are still needed.

A different pattern of changes was observed in the expression of xc- after CEF administration. Compared with sham-operated rats, CEF treatment resulted in an upregulation of mRNA expression in the dorsal striatum, but protein levels increased only in the frontal cortex. In animals subjected to a 90 min MCAO after CEF, increased levels of xc- mRNA were found in the frontal cortex compared with the non-treated group. However, significantly lower amounts of mRNA in the hippocampus and striatum were detected in pretreated rats. Given that xc- exports Glu from cells, and in ischemia, excitotoxicity-dependent injury is associated with a dramatic rise of extracellular Glu (Lipton 1999), this downregulation of xc- expression could be protective. Moreover, in response to cerebral ischemia, microglial and astroglial cells are activated, and it is known that activated microglia and astrocytes release Glu via xc- that can kill neurons (Piani and Fontana 1994; Fogal et al. 2007).

NAC administration caused different effects than CEF on the Glu transporters investigated. In non-ischemic rats, we observed lower levels of GLT-1 mRNA in the hippocampus, lower GLT-1 protein in the hippocampus and cortex and a lack of significant changes in the dorsal striatum. Similarly, Knackstedt et al. (2010) analyzed GLT-1 protein expression after chronic NAC treatment and did not observe any changes in the nucleus accumbens (ventral striatum). Additionally, in R6/1 mice, NAC failed to influence GLT-1 expression in the cortex and striatum (Wright et al. 2015). In animals subjected to a 90 min MCAO, pretreatment with NAC resulted in decreased GLT-1 mRNA expression only in the striatum with no significant changes in protein levels, which differs from the findings on CEF-induced effects. There is a general agreement that GLT-1 is important in the induction of brain tolerance, but results of published studies are inconsistent. Several reports describe an increase in GLT-1 expression after preconditioning stimulus (Pradillo et al. 2006; Romera et al. 2007; Zhang et al. 2007; Bigdeli et al. 2008), but there are also data suggesting that GLT-1 downregulation is crucial to induce brain tolerance (Douen et al. 2000; Kosugi et al. 2005). Moreover, results of several studies have suggested that an excess of extracellular Glu after ischemia reversed the mode of action of GLT-1 and subsequently augmented tissue damage (Douen et al. 2000; Kosugi et al. 2005; Verma et al. 2010). Thus, downregulation of GLT-1 expression could be protective. Since NAC, among its various mechanisms of action, also inhibits NFκB activation (Schreck et al. 1992), which can induce GLT-1 expression, the observed downregulation of GLT-1 expression could be partially driven by NFκB activity. However, differences between the examined cerebral regions raise a question about the area-specific distribution and regulation of transporter expression.

NAC injection, both in ischemic and non-ischemic rats, caused a significant drop in xc- protein levels in investigated regions. As discussed above, the downregulation of this transporter could be protective via a subsequent decrease in Glu export from the cells, thereby decreasing the extracellular Glu concentration, which plays a deleterious role in cerebral ischemia.

Interestingly, two of the other preconditioning methods investigated, i.e., IP and 3NP, exerted identical effects on the expression levels of GLT-1 among rats that were not subjected to prolonged ischemia. IP and 3NP caused a significant downregulation of GLT-1 mRNA levels in the frontal cortex and hippocampus; however, GLT-1 protein levels remained unchanged. After a subsequent 90 min MCAO, the observed response resembled that found after CEF pretreatment, i.e., higher levels of mRNA in the frontal cortex and higher protein levels in all three of the examined structures. These results suggest that GLT-1 plays an important protective role in the development of brain tolerance to ischemia. Similar effects of IP in focal cerebral ischemia were reported by Bigdeli et al. (2009). To the best of our knowledge, the influence of 3NP on GLT-1 in the context of brain tolerance has not been reported. However, it should be noted that GLT-1 expression levels after a 90 min MCAO preceded by preconditioning were comparable with the SHAM group levels. Therefore, the GLT-1 protein levels could be related to the preservation of cellular sources in the rescued regions (i.e., the frontal cortex and hippocampus) and unsuccessful preconditioning within the infarction core (dorsal striatum). The identical effects of 3NP and IP on GLT-1 expression was consistent with their similar influence on the expression of proinflammatory cytokines such as IL-1β and TNF-α, which are also regulated by NFκB (Pera et al. 2004), a well-recognized modulator of GLT-1 expression (Takahashi et al. 2015).

3NP and IP exerted similar effects on xc- mRNA levels but not on protein levels. Compared with SHAM rats, preconditioning alone caused a decrease in the amount of mRNA in the frontal cortex and hippocampus, which mirrored changes observed in GLT-1 expression. Additionally, protein levels did not change significantly. However, in animals subjected to a 90 min MCAO, a prior application of 3NP or IP resulted in increased mRNA levels in the cortex and hippocampus, whereas protein levels were lower in the striatum in 3NP rats and higher in all three regions after IP. Moreover, this protein increase in the frontal cortex exceeded the levels measured in the SHAM animals. These results suggest that xc- is one of the players in the protection caused by IP. What the underlying mechanism is remains unclear. To the best of our knowledge, this is the first study that has investigated the association between preconditioning with 3NP/IP and xc- expression in focal brain ischemia. Sims et al. (2012) showed that short-term hypoxia used as a preconditioning stimulus upregulated xc- mRNA and protein expression and changed xc- activity in the brain in C57BL/6 mice. In hypoxic and IP, low-degree oxidative stress plays an important role in the activation of the nuclear factor erythroid 2-related factor 2 (Nrf-2) (Thompson et al. 2012), which can induce xc- expression (Massie et al. 2015). Nrf-2 is also implicated in CEF-related neuroprotection involving xc- (Lewerenz et al. 2009). However, in the current study, the effects of CEF and IP on xc- expression differed. In addition, preconditioning with 3NP is associated with oxidative stress and reactive oxygen species generation (Wiegand et al. 1999). In contrast to IP rats, we failed to detect any changes xc-protein levels after a 90 min MCAO, although both 3NP and IP showed similar effects on mRNA expression. Altogether, these observations strongly suggest that the induction of brain tolerance to ischemia is a very complex process and that different preconditioning stimuli can evoke different effects on particular pathways and molecules.

In summary, in the current study, we found that all four of the tested strategies, i.e., CEF, NAC, 3NP, and IP, induced brain tolerance to prolonged focal ischemia. Pharmacological preconditioning with CEF and NAC showed similar neuroprotective effects, measured as infarct volume and neurological deficit, as two established strategies. All of these strategies influenced the expression of the Glu transporters investigated, suggesting that they play an important role in the induction of brain tolerance. However, the effects differed among the preconditioning stimuli. CEF, 3NP, and IP upregulated GLT-1 protein levels after prolonged ischemia, whereas GLT-1 appears not to be the key target of action of NAC. Instead, after NAC preconditioning, we found a significant downregulation of xc- protein in ischemic brains. Interestingly, IP prior to a 90-min MCAO resulted in an increased expression of this transporter.

## Conclusions

In summary, CEF, NAC, 3NP, and IP showed neuroprotective effects after focal brain ischemia, indicating similar reductions in neurological deficits. These effects were associated with changes in the expression levels of two glutamatergic system transporters, GLT-1 and xc-.
